# Risk Factors for Sporadic Pancreatic Neuroendocrine Tumors: A Case-Control Study

**DOI:** 10.1038/srep36073

**Published:** 2016-10-26

**Authors:** Qiwen Ben, Jie Zhong, Jian Fei, Haitao Chen, Lifen Yv, Jihong Tan, Yaozong Yuan

**Affiliations:** 1 Department of Gastroenterology, Ruijin Hospital, Shanghai Jiaotong University, Shanghai, 200025, China; 2 Department of General Surgery, Ruijin Hospital, Shanghai Jiaotong University, Shanghai, 200025, China; 3 Department of Geriatrics, Changhai Hospital of Second Military Medical University, Shanghai, China

## Abstract

The current study examined risk factors for sporadic pancreatic neuroendocrine tumors (PNETs), including smoking, alcohol use, first-degree family history of any cancer (FHC), and diabetes in the Han Chinese ethnic group. In this clinic-based case-control analysis on 385 patients with sporadic PNETs and 614 age- and sex-matched controls, we interviewed subjects using a specific questionnaire on demographics and potential risk factors. An unconditional multivariable logistic regression analysis was used to estimate adjusted odds ratios (AORs). No significant differences were found between patients and controls in terms of demographic variables. Most of the patients with PNETs had well-differentiated PNETs (G1, 62.9%) and non-advanced European Neuroendocrine Tumor Society (ENETS) stage (stage I or II, 83.9%). Ever/heavy smoking, a history of diabetes and a first-degree FHC were independent risk factors for non-functional PNETs. Only heavy drinking was found to be an independent risk factor for functional PNETs (AOR = 1.87; 95% confidence interval [CI], 1.01–3.51). Ever/heavy smoking was also associated with advanced ENETS staging (stage III or IV) at the time of diagnosis. This study identified first-degree FHC, ever/heavy smoking, and diabetes as risk factors for non-functional PNETs, while heavy drinking as a risk factor for functional PNETs.

Gastroenteropancreatic neuroendocrine tumors (GEP-NETs) arise from the diffuse neuroendocrine system that are capable of producing biogenic amines and polypeptide hormones^1^. Approximately 4–7% of GEP-NETs are located in the pancreas, referred to as pancreatic neuroendocrine tumors (PNETs)^2^, which account for 1.3–2.8% of new pancreatic neoplasms by incidence. Although PNETs are generally considered rare, with an incidence of approximately 0.3 per 100,000 people in the Western population^3^,^4^ and 1.01/100,000 in Japan^5^, their incidence is significantly increasing owing to improvements in diagnosis and case finding^6^. Among all GEP-NETs, PNETs represent the worst prognosis because most of them present with metastasis disease at the time of diagnosis and are not surgically treatable.

The majority of PNETs is sporadic, although PNETs can be observed in relation to inherited syndromes such as von Hippel-Lindau (VHL) disease^7^ and multiple endocrine neoplasia type 1 (MEN1)^8^. Many epidemiological studies have evaluated risk factors for pancreatic adenocarcinomas^9^,^10^,^11^; however, little is known about the possible role of environmental risk factors for sporadic PNETs^12^. Several potential risk factors have been postulated for PNETs, which include social economic factors, family history of cancer (FHC), smoking habits, alcohol consumption, body mass index (BMI) and type 2 diabetes^13^,^14^,^15^,^16^,^17^. All of these studies have a case-control design and have been conducted in the USA^13^,^14^,^17^, Europe^15^ and China^16^. Hassan *et al*. conducted a hospital-based case-control study involving 160 PNETs cases, and observed a significant relationship between a first-degree family history of esophageal cancer and increased risk of PNETs (adjusted odds ratios [AORs] = 5.6; 95% confidence intervals [CIs], 1.1–29.6)^13^. However, this study included patients with hereditary PNET (MEN-1), which could have influenced the risk estimation. Based on a case-control study from Italy comprising 162 sporadic PNETs and 648 controls, Capurso *et al*. found that a first-degree FHC (OR = 2.2; 95% CI, 1.5–3.2), high alcohol intake (OR = 4.8, 95% CI, 2.4–9.5), history of chronic pancreatitis (OR = 8.6; 95% CI, 1.4–51) and recent-onset diabetes (OR = 40.1; 95% CI, 4.8–328.9) were all independent risk factors for the development of PNETs^15^.

The aim of our study was to complete a large case-control study of sporadic PNETs in the Han Chinese ethnic group and evaluate information on a variety of potential exposures related to PNETs risk, including a history of diabetes, alcohol consumption, smoking habits, and a first-degree FHC. Because functional (F) and non-functional (NF)-PNETs show different clinical behavior and prognosis, we present results for F- and NF-PNETs combined and separately.

## Results

### Patient characteristics

Three hundred and eighty-five patients with PNETs were age and sex matched with 614 control subjects, with the ratio of 1:1.59. The distribution of demographic features of cases and controls is shown in Table 1. The mean age (±SD) was 49.7 (±11.8) years for PNET patients and 48.5 (±9.4) years for controls. Most of the patients and controls were female (55.1% and 55.2%, respectively), lived in urban areas (80.8% and 77.7%, respectively), and were educated up to middle or high school level (56.1% and 60.1%, respectively). No statistically significant differences were found between patients and controls in terms of these variables, suggesting that the frequency matching was adequate.

Table 2 summarized the clinical features of the 385 PNET patients. There were 142 (36.9%) non-functional and 243 (63.1%) functional tumors. Most (58.4%) of the functional PNETs were insulinomas. Most of the patients had well-differentiated endocrine tumors (G1, 62.9%). With respect to ENETS staging, 217 patients (56.4%) were in stage I, 65 patients (16.9%) were in stage IIa, and 41 patients (10.6%) were in stage IIb. Twelve patients with tumor-invading adjacent structures were defined as stage IIIa. Lymph nodes were involved in 27 cases (23.6%) that were defined as stage IIIb. Twenty-three patients (6.0%) who had distant metastases at diagnosis were defined as stage IV.

### Risk factors for total PNET

As shown in Table 3, unconditional logistic regression analysis was used to estimate risk associations between different factors and risk of total PNET (including functional and non-functional PNETs). The univariate analyses indicated that heavy alcohol consumption, ever/heavy smoking, and first-degree FHC (yes vs. no) were significant risk factors for PNET, whereas ever alcohol drinking, regions and educational levels were not significant factors. Multivariable analyses with adjustments for risk factors showed that ever/heavy cigarette smoking and first-degree FHC were independently associated with PNET risk, with multivariate AORs (95% CIs) of 1.60 (1.10–2.33) for ever smoking, 2.07 (1.15–3.73) for heavy smoking, and 1.60 (1.01–2.40) for first-degree FHC. Furthermore, heavy drinking (≥30 g/day) was not associated with higher risk of PNETs development in the multivariate model (AORs = 1.31; 95% CIs, 0.74–2.31).

### Risk factors for F-NET

Because functional and non-functional tumors have evidently different clinical behavior and outcome, we conducted analyses restricting to F- (n = 243) and NF- PNET (n = 142), respectively. Univariate analysis indicated that regions, educational levels, ever alcohol drinking and first-degree FHC were not significant risk factors for the development of F-NET, whereas heavy alcohol consumption (≥30 g/day) and ever/heavy smoking were significant risk factors for F-PNETs. Multivariable analyses showed that only heavy alcohol use was independently associated with F-PNET risk, with multivariate AORs (95% CIs) of 1.87 (1.01–3.51; Table 3).

### Risk factors for NF-PNET

In the analysis of NF-PNET, in addition to the variables of regions, educational levels, alcohol consumption, smoking status, and first-degree FHC, we also included a history of diabetes as a potential risk factor. Multivariable analyses with adjustments for risk factors showed that ever/heavy smoking, first-degree FHC and a history of diabetes were independent risk factors for NF-PNETs, with multivariate adjusted ORs (95% CIs) of 1.52 (1.01–2.39) for ever smoking, 1.86 (1.23–3.43) for heavy smoking, 1.93 (1.14–3.25) for first-degree FHC and 1.96 (1.14–3.70) for a history of diabetes. However, ever/heavy alcohol use was not associated with the risk of NF-PNETs. In addition, multivariable analysis with adjustments for ever smoking and first-degree FHC showed that new-onset diabetes (≤1 year) was significantly associated with development of NF-PNETs, with an adjusted OR(95% CI) of 2.67(1.37–5.20; Table 3).

### Risk factors and WHO classification and ENETS stage

As shown in Table 4, we compared PNET patients with a well WHO classification at the time of diagnosis (G1, n = 242) and those with a poor or moderate classification (G2 + G3, n = 111). None of the risk factors (regions, educational levels, ever or heavy alcohol consumption, ever or heavy smoking, first degree FHC) were associated with the WHO classification. In the analysis restricting to F- and NF- PNET, respectively, we found that none of the risk factors were associated with the WHO classification (P > 0.05; Table 4).

We then assessed whether any of the identified risk factors were correlated with a more advanced ENETS staging (TNM stage III or IV). The following risk factors—regions, educational levels, alcohol consumption (ever or heavy), first degree FHC, and a history of diabetes—were not associated with ENETS staging (P > 0.05). Interestingly, ever (P = 0.014) and heavy smokers (P = 0.003) were more likely to be diagnosed with advanced ENETS staging than never smokers. Furthermore, in the analysis restricting to NF- PNET, we found that ever smokers (P = 0.035) and heavy smokers (P = 0.002) were more likely to be diagnosed as having advanced ENETS staging than never smokers. When performing the analysis restricting to F- PNET, none of the risk factors were associated with the advanced ENETS staging (TNM stage III or IV).

Additionally, we evaluated whether the effects of cigarette smoking on the ENETS staging were independent of regions and educational levels (Supplementary Table 1). For NF-PNET, neither regions nor educational levels were associated with ENETS staging in ever/heavy smokers (P > 0.05). Similar results were also shown for total PNET.

## Discussion

Unlike the studies evaluating risk factors associated with exocrine pancreatic carcinomas, risk factors to date have not been systematically identified for PNETs. In this large hospital-based case-control study, we found independent associations between ever/heavy smoking, first-degree FHC, a history of diabetes and the risk of NF-PNETs. However, only heavy drinking was indicated to be independently associated with the development of F-PNETs. Interestingly, ever/heavy smoking was associated with advanced ENETS staging in NF-PNETs.

Although smoking is clearly one of the most preventable causes of pancreatic carcinoma development^18^,^19^, little is known about the role of smoking in the development of PNET. Several recently conducted case-control studies showed no positive association between ever smoking and the development of NETs in the pancreas^14^, rectum^20^ and small intestine^21^. In another research from Italy found that although heavy smoking was associated with a slightly increased risk of PNET (OR = 1.5; 95% CI: 1–2.4) in the univariate analysis, neither smoking nor heavy smoking was associated with an increased risk in the multivariate analysis. Results of our analyses indicated that ever/heavy smoking were related to an elevated risk of NF-PNETs in the multivariate analysis (ever smoking: OR = 1.52, 95%CI: 1.01–2.39, P = 0.046; heavy smoking: OR = 1.86, 95%CI: 1.23–3.43, P = 0.018). However, we did not find a significant association between ever/heavy smoking and F-PNET in the multivariable models, although univariable models indicated a significant association. Our research suggests that the tumorogenesis of NF-PNET was different from that of F-PNET. The effects of smoking on the risk of PNETs remain uncertain and merit further study.

The association between diabetes and pancreatic carcinoma has been well examined^10^,^22^,^23^,^24^. With respect to the association between diabetes and PNETs risk, several studies^14^,^15^,^17^ including two meta-analyses^12^,^25^ have consistently indicated diabetes as a potential risk factor for development of PNETs. Hassan *et al*. studied 160 patients with PNETs in their case-control study and observed that diabetes was associated with significantly increased risk of PNETs (OR = 2.8; 95% CI, 1.5–5.2)^14^. These results were supported by two independent studies from Capurso *et al*.^15^ and Halfdanarson *et al*.^17^ Similarly, our data provided strong evidence of an association between diabetes and the risk of NF-PNET, with an AOR of 1.96 (95% CI, 1.14–3.70). Because most functional tumors in this study were insulinomas (92.6%), which are scarcely diagnosed as diabetes^26^, we evaluated the role of diabetes only in the development of NF-PNETs. In addition, our study excluded all patients with an inherited syndrome (MEN-1 and VHL). One earlier study was conducted on cases diagnosed as either F-PNETs or NF-PNETs^15^, and two other studies did not describe the biological behavior of the tumors^14^,^17^. The mechanisms linking diabetes to PNETs development remain unknown. Several studies hypothesized that a family history of MEN-1^27^ and the presence of a glucagon-producing tumor originating from the alpha cells of the pancreas^28^ may result in elevated blood glucose levels. Furthermore, it is possible that diabetes may act as a mediator for chronic inflammation and oxidative stress inside the cell, which may lead to DNA mutation and the development of PNETs^29^,^30^.

Two previous case–control studies investigated the association between diabetes duration and PNET risk^14^,^15^. The effect estimate for subjects with recent onset (≤1 year) diabetes was higher (OR 12.80, 95%CI 2.47–66.42) than those with long-standing (>1 years) diabetes^25^. In line with these results, our research indicated recent onset (≤1 year) diabetes (but not long duration of diabetes) was related to elevated risk of NF-PNET after adjustments for ever smoking and first degree FHC. This time-course characteristic strongly supported the hypothesis that DM might also be a consequence of NF-PNET, similar to the association between diabetes and pancreatic cancer^10^,^22^. The underlying mechanisms by which NF-PNET leads to DM might depend on the destruction of pancreatic beta cells and the development of peripheral insulin resistance^31^. Given the low rate of PNET, large multicenter studies would be necessary to explore the association between DM and NF-PNET.

In line with previous reports^14^,^15^,^16^, we observed the strong association between first-degree FHC and risk of NF-PNETs development, which was similar to the results for pancreatic carcinoma^32^,^33^,^34^. The increased risk of NF-PNET in subjects with a family history of cancer may be due to unknown genetic factors and shared environmental factors^13^,^35^,^36^. Several case-control studies have identified a possible role for apoptosis and inflammatory pathways in the etiology of NET, such as variants of the tumor necrosis factor alpha gene, interleukin 2 gene, and defender against cell death gene^35^,^36^,^37^.

Results from our study indicate no associations between ever/heavy alcohol drinking and NF-PNETs risk, which was comparable to the reports from Hassan *et al*.^14^, but was in contrast to the other reports^15^,^17^. We believe this inconsistency could be attributed to the limited size of the study sample, the different inclusion criteria for cases, and the different methods of quantification of alcohol consumption. In addition, we observed an independent association between heavy drinking and development of F-PNET, which was in line with the results from Zhan *et al*.^16^.

Only one study^15^ presented data on the influence of risk factors on PNET patients’ progression and outcome. In that report, the authors observed an association between history of diabetes and metastatic disease at the time of diagnosis (P = 0.012). No other factors were related to more aggressive disease features^15^. Interestingly, our data showed that in patients with NF-PNETs, an increased prevalence of advanced ENETS staging (stage III or IV) was associated with ever/heavy smoking (P < 0.05), but not with regions, educational levels, a history of diabetes, ever/heavy alcohol use and first degree FHC (P > 0.05). Tumors may be diagnosed at a more advanced stage in individuals who have a less favorable economic/cultural level or a “less healthy” behavior, because they may not urge to report symptoms early. In the present data, we further evaluated whether the effects of cigarette smoking on the ENETS staging were independent of regions and educational levels. Our data indicated that neither regions nor cultural levels were associated with ENETS staging in ever/heavy smokers (P > 0.05).

Research has shown genetic alterations in the lung epithelium of smokers, and increased microsatellite instability in colon tumors of smokers^38^,^39^. Cigarette smoke contained several carcinogens, which may reach the pancreas from the bloodstream and refluxed bile, suggesting the potential mechanism linking smoking to the development of pancreatic tumor^40^,^41^. Momi *et al*. observed nicotine/cigarette smoke promotes metastasis of pancreatic cancer through alpha-7nACh mediated MUC4 upregulation^41^. A recent study by Delitto *et al*. showed that nicotine reduced pancreatic cancer survival via augmentation of paracrine hepatocyte growth factor/c-MET signaling in the tissue microenvironment^40^. However, mechanisms linking smoking to NF-PNET progression have not been explored, which should be examined in the future.

To the best of our knowledge, this case-control study is the largest to assess several risk factors for PNETs with proper adjustment for potential risk factors. The diagnosis was confirmed in each patient by individually reviewing pathology slides and reports to ensure that diagnostic inclusion criteria were met. Importantly, we explored the potential risk factors for F-PNET and NF-PNET, respectively, given the differences in clinical behavior and prognosis between the two disease entities.

We acknowledge that our study has certain limitations. First, the possibility of selection bias owing to its population (hospitalized patients with PNETs) and design (retrospective data reviewing) cannot be ruled out. Nevertheless, because both cases and controls belonged to a relatively homogeneous base population and were matched by sex, age, and sociodemographic variables, we believe that the bias would be minimized. In addition, given the rarity of PNETs and the need for confirmed pathologic diagnosis, our approach of retrospective data collection was appropriate.

Second, many potentially confounding factors could not be addressed owing to no established risk factors for PNETs. We specifically considered the association for risk factors in exocrine pancreatic cancer in the model but could not examine the influence of numerous host and/or environmental factors on risk, including chronic pancreatitis, allergies, BMI, H pylori infection, dietary factors, and commonly prescribed medications (use of statins, aspirin and hypoglycemic agents *et al*.). For example, the effect of obesity on the development of PNETs cannot be excluded, as obesity may be associated with type 2 diabetes development in patients with PNETs. Unfortunately, the patient records in our database contained no information about the patients’ BMI before the diagnosis of PNETs. The baseline BMI may not have accurately reflected the patients’ obesity history, because some PNETs patients experienced disease-related weight loss or weight gain. We did not consider the potential effects of commonly prescribed medications, such as use of statins, aspirin, which were reported to be inversely associated with risk of exocrine pancreatic cancer. Again, data on use of hypoglycemic agents (such as metformin, thiazolidinediones, insulin *et al*.) were not available in most of those diabetic individuals. Many researches, to date, have suggested that metformin and thiazolidinediones could exert a protective role against the development and progression of some cancers^42^,^43^, whereas insulin was associated with an increased risk of cancer 44,45.

Third, the potential measurement errors could not be excluded when assessing risk factors. For instance, ever smokers/drinkers may include individuals with low level substance abuse who quit several years ago as well as patients who were heavy users and quit more recently. Furthermore, it was difficult to distinguish between type 1 and type 2 diabetes in most of our diabetic subjects. However, it was likely that the majority of diabetic individuals had type 2 diabetes because it is late onset and received treatments with only oral hypoglycemic agents.

Owing to the popularity of the endocrinology department at Ruijin Hospital of Shanghai Jiaotong University, patients with F-PNET were higher in our series than those in other studies (63.1% vs. 19.1%)^15^. We are continuing to establish a national consortium to assist in the development of a large multicenter epidemiologic study in China to examine several environmental, social, behavioral, occupational, and genetic risk factors and to assess gene-environment interactions in GEP-NETs^46^.

In summary, our study shows the different risk factors between F- and NF-PNET, suggesting different biological behavior and clinical characteristics between the two disease entities. Ever/heavy smoking and histories of diabetes and first-degree FHC may be potential risk factors for NF-PNETs, while heavy drinking may be one of the risk factors for F-PNETs. In addition, prediagnosis ever/heavy smoking may be associated with advanced ENETS staging (stage III or IV) in NF-PNETs. Although prospective studies are needed to validate these results, our preliminary findings may provide guidance in the development of PNETs surveillance programs in the future.

## Material and Methods

### Study design

The study design was an ongoing hospital-based case-control study conducted at Ruijin Hospital, Shanghai, People’s Republic of China. The purpose of the study was to examine risk factors that contribute to the development of PNETs. The Ethics Committee of Ruijin Hospital approved the study protocol. The methods were carried out in accordance with the approved guidelines. After written informed consent was obtained, each participant was scheduled for an interview by using a structured questionnaire to collect demographic and exposure information.

### Cases

**Figure 1:**
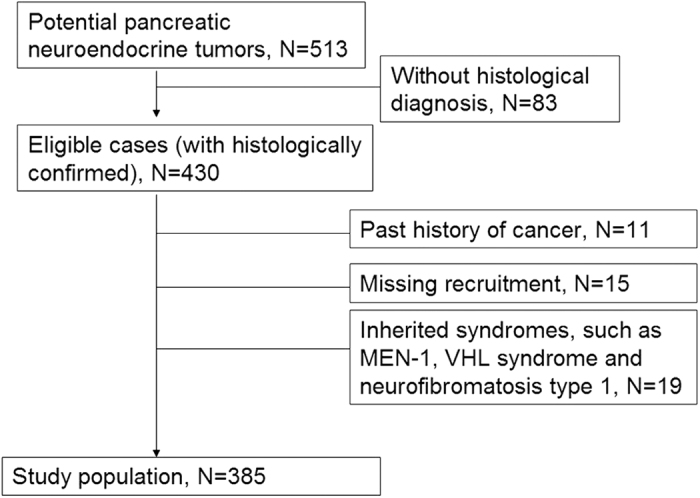
Flowchart of patient selection.

Patients eligible for this study were enrolled between January 1, 2001 and June 30, 2015. There were 513 potential PNETs patients and 430 patients with pathologically confirmed primary PNETs during the study period. Of these, 45 cases were excluded, because 11 had a history of cancer, 15 missed recruitment, and 19 patients had a clinical diagnosis of inherited syndromes such as MEN-1, VHL syndrome, and neurofibromatosis type 1. The remaining 385 patients were enrolled in this study (Fig. 1).

Features of the tumors (size, location, lymph invasion and distant metastasis, mitotic count, Ki-67, etc.) were all based on intraoperative findings and pathological analysis. The WHO (World Health Organization) 2010 classification was used to classify PNETs as well-differentiated endocrine tumors (G1: mitotic count ≤2/10 HPF, Ki-67 ≤2%), well-differentiated endocrine carcinomas (G2: mitotic count 2–20/10 HPF, Ki-67 3–20%), or poorly differentiated endocrine carcinomas (G3: mitotic count >20/10 HPF, Ki-67 >20%)^47^. The tumor-node-metastasis (TNM) stage of the European Neuroendocrine Tumor Society (ENETS)^48^ was used to stratify PNET stage at diagnosis. In addition to the standard panel of markers of neuroendocrine differentiation (synaptophysin, chromogranin, and neuron-specific enolase), immunohistochemistry (IHC) included staining for insulin, glucagon, somatostatin, pancreatic polypeptide, gastrin, and vasoactive intestinal peptide. The tumors were classified as clinically functioning PNET (F-PNET), if symptoms and circulating levels attributable to the corresponding peptide were concordant with immunostaining.

### Controls

Subjects who were diagnosed with nonmalignant disease (including those with gallbladder polyps, polycystic kidney, breast fibroadenoma, uterine fibroids) based on discharge diagnoses in the same hospital during the same period were included as the controls. Eligible controls were age- (in 3-year age groups) and sex-matched inpatients, and underwent imaging tests and tumor marker tests (including CA19-9, CEA, AFP, *etc.*) to exclude potential asymptomatic common tumors. Patients with a history of malignant disease or having received any cytotoxic treatment were excluded. Conditions related to alcohol and tobacco consumption (e.g., respiratory diseases, peptic ulcer, and hepatic disease) or any chronic diseases (e.g., diabetes, cardiovascular disease) that might have resulted from substantial lifestyle modifications were excluded. Informed consent was obtained from all patients. After screening, we included 614 controls.

### Data collection

Cases and controls were personally interviewed for demographic characteristics (age, sex, educational level, and region); prediagnostic personal habits (smoking status and alcohol drinking); and histories of diabetes mellitus and first-degree FHC. Participants were classified as “ever-smokers” if they reported having smokers more than 100 cigarettes during their lifetime. Accordingly, never smokers were defined if they smoked less than 100 cigarettes during their lifetime. Smoking amount was recorded in terms of pack-years (pack-year = numbers of packs of cigarettes/day × years of smoking). Heavy smokers were classified if they had smoked for ≥21 pack-years, respectively^15^. Participants were classified as “ever-drinkers” if they had consumed >1 serving/day (12.5 g/day) of alcoholic beverage (beer, wine or liquor) for a duration of at least 6 months^49^. For each beverage type, participants were asked to recall the number of drinks they typically consumed each week and the number of years during which they consumed that beverage. These answers were integrated into a scoring system that was used to classify alcohol consumption as “heavy drinking” (≥30 g/day)^50^,^51^. Diabetes was defined as present if the fasting serum glucose level was greater than 7.0 mmol/L or a previous diagnosis of diabetes mellitus was made based on the American Diabetes Association criteria^52^. The course of DM was calculated from the date of diagnosis of DM to the date of PNET diagnosis. As the previous published studies^14^,^15^, duration of DM was dichotomized at 1 year to define cases of DM as new-onset or long standing DM. For those diagnosed on admission, the course was recorded as less than 1 year.

### Statistical analysis

All statistical analyses were conducted using the SPSS 19.0 statistical software program (SPSS, Chicago, IL, USA). All tests were two-tailed, and a *P* value of <0.05 was considered to indicate statistical significance. Pearson’s *χ*^2^ test (Fisher’s exact test) was used to compare the sociodemographic and clinicopathologic data. Crude and adjusted OR and 95% CI for each variable were calculated by using unconditional logistic regression analysis. Potential confounders were included in the multivariate analysis in a stepwise manner at a significance level of *P* < 0.15. We divided the PNETs cases as functional and non-functional (NF) tumor, because both show different clinical behavior and prognosis. For functional PNETs, equations included terms for a first-degree FHC, smoking status, and alcohol drinking. For NF-PNETs, we also included a history of diabetes in addition to the above three variables.

## Additional Information

**How to cite this article**: Ben, Q. *et al*. Risk Factors for Sporadic Pancreatic Neuroendocrine Tumors: A Case-Control Study. *Sci. Rep.* **6**, 36073; doi: 10.1038/srep36073 (2016).

**Publisher’s note:** Springer Nature remains neutral with regard to jurisdictional claims in published maps and institutional affiliations.

## Supplementary Material

Supplementary Information

## Figures and Tables

**Table 1:** Demographic features of the Study Population.

Variables	Cases, n = 385(%)	Control, n = 614(%)	P*
**Age** (Mean ± SD)	49.7 ± 11.8	48.5 ± 9.4	
≤40	83 (21.6)	125 (20.4)	0.712
40–50	126 (32.7)	183 (29.8)	
50–60	92 (23.9)	156 (25.4)	
60–70	68 (17.7)	116 (18.9)	
≥70	16 (4.2)	34 (5.5)	
**Gender**			0.964
Men	173 (44.9)	275 (44.8)	
Women	212 (55.1)	339 (55.2)	
**Region**			0.244
Urban area	311 (80.8)	477 (77.7)	
Rural area	74 (19.2)	137 (22.3)	
**Education levels**			0.802
Elementary school or less	13 (3.4)	25 (4.1)	
Middle or high school	216 (56.1)	369 (60.1)	
College or higher level of education	142 (36.9)	184 (30.0)	
Missing data	14 (3.6)	36 (5.9)	

Note: *Pearson’s χ2 test.

**Table 2:** Clinical features of the Pancreatic Neuroendocrine Tumors case.

Variables	Cases, n = 385	%
Clinical functioning		
Nonfunctioning	142	36.9
Functioning	243	63.1
Insulinoma	225	58.4
Gastrinoma	8	2.1
Glucagonoma	7	1.8
VIPoma	3	0.8
WHO Classification		
NET G1	242	62.9
NET G2	85	22.1
NEC G3	26	6.8
Missing data	32	8.3
ENETS Stage at diagnosis		
I	217	56.4
II a	65	16.9
II b	41	10.6
III a	12	3.1
III b	27	7.0
IV	23	6.0

**Table 3:** Pancreatic Neuroendocrine Tumors Risk Factors: Univariate and Multivariate Logistic Regression Analyses.

NF- and F-PNET	Casesn = 385	Controlsn = 614	Univariate OR (95% CI)	P	Multivariate OR (95% CI)*	P
Region						
Urban area	311	477	1.0			
Rural area	74	137	0.83 (0.60–1.14)	0.244	—	
Education levels						
Elementary school or less	13	25	1.0			
Middle or high school	216	369	1.13 (0.56–2.25)	0.730	—	
College or higher	142	184	1.48 (0.73–3.00)	0.277	—	
Alcohol drinking						
Never	329	534	1 (reference)		1 (reference)	
Ever	56	80	1.14 (0.79–1.64)	0.479	—	
Heavy drinking	34	30	1.84 (1.11–3.06)	0.019	1.31 (0.74–2.31)	0.359
Smoking status						
Never	295	515	1 (reference)		1 (reference)	
Ever	90	99	1.57 (1.14–2.16)	0.006	1.60 (1.10–2.33)	0.014
Heavy smoking	44	39	1.96 (1.24–3.09)	0.004	2.07 (1.15–3.73)	0.015
First degree FHC						
No	331	559	1 (reference)		1 (reference)	
Yes	54	55	1.66 (1.11–2.47)	0.013	1.60 (1.07–2.40)	0.023
F-PNET	243	614				
Region						
Urban area	199	477	1.0			
Rural area	44	137	0.77 (0.53–1.12)	0.174	—	
Education levels**						
Elementary school or less	7	25	1.0			
Middle or high school	159	369	0.65 (0.28–1.53)	0.322	—	
College or higher	65	184	0.79 (0.33–1.92)	0.606	-	
Alcohol drinking						
Never	207	534	1 (reference)		1 (reference)	
Ever	36	80	1.16 (0.76–1.78)	0.491	—	
Heavy drinking	27	30	2.32 (1.35–4.00)	0.002	1.87 (1.01–3.51)	0.048
Smoking status						
Never	190	515	1 (reference)		1 (reference)	
Ever	53	99	1.43 (0.99–2.08)	0.058	1.35 (0.85–2.13)	0.204
Heavy smoking	26	39	1.80 (1.07–3.04)	0.027	1.43 (0.66–3.10)	0.361
First degree FHC						
No	214	559	1 (reference)		1 (reference)	
Yes	29	55	1.38 (0.93–2.12)	0.121	1.31 (0.80–2.13)	0.281
NF -PNET	n = 142	n = 614				
Region						
Urban area	112	477	1.0			
Rural area	30	137	0.93 (0.60–1.46)	0.759	—	
Education levels**						
Elementary school or less	6	25	1.0			
Middle or high school	57	369	1.55 (0.61–3.95)	0.352	—	
College or higher	77	184	0.57 (0.23–1.45)	0.236	—	
Alcohol drinking						
Never	122	534	1 (reference)		1 (reference)	
Ever	20	80	1.09 (0.65–1.86)	0.738	—	
Heavy drinking	7	30	1.02 (0.44–2.38)	0.961	—	
Smoking status						
Never	105	515	1(reference)		1(reference)	
Ever	37	99	1.81 (1.18–2.79)	0.007	1.52 (1.01–2.39)	0.047
Heavy smoking	18	39	2.24 (1.23–4.06)	0.008	1.86 (1.23–3.43)	0.018
First degree FHC						
No	117	559	1 (reference)		1 (reference)	
Yes	25	55	2.17 (1.30–3.63)	0.003	1.93 (1.14–3.25)	0.014
Diabetes mellitus						
No	118	564	1 (reference)		1 (reference)	
Yes	24	50	2.29 (1.36–3.88)	0.002	1.96 (1.14–3.70)	0.016
Duration, years						
≤1	15	28	2.56 (1.37–5.20)	0.005	2.67 (1.37–5.20)	0.004
>1	9	22	1.96 (0.88–4.35)	0.101	1.23 (0.54–2.89)	0.631

^*^Adjusted by smoking status, alcohol drinking, a first degree family history of any cancer and a history of diabetes (in the analysis for NF-PNET).

^**^Data on education were not available in 12 patients with F-PNET and 2 patients with NF-PNET, respectively.

**Table 4:** Distribution of risk factors in patients with sporadic pancreatic endocrine tumors according to their ENETS staging and WHO classification at the time of diagnosis

PNET	ENETS staging				WHO classification			
	n	I + II (n = 323)	III + IV (n = 62)	P#	n**	G2 + G3 (n = 111)	G1 (n = 242)	P#
Urban area, yes	311	261	50	0.977	305	95	210	0.762
Middle school or higher, yes	358	301	57	0.723	335	104	231	0.313
Alcohol drinking, ever	56	45	11	0.436	50	15	35	0.812
Heavy drinking	34	28	6	0.798	29	12	17	0.229
Smoking status, ever	90	68	22	**0.014**	82	29	53	0.383
Heavy smoking	44	30	14	**0.003**	41	17	24	0.142
First degree FHC	54	44	10	0.603	46	17	29	0.388
**NF-PNET**	**n**	**I** + **II (n** = **93)**	**III** + **IV (n** = **49)**	**P**#	n**	**G2** + **G3 (n** = **45)**	**G1 (n** = **89)**	**P**#
Urban area, yes	112	72	40	0.559	117	38	79	0.478
Middle school or higher, yes	134	90	44	0.084	126	42	84	0.193
Alcohol drinking, ever	20	13	7	0.960	18	7	11	0.608
Heavy drinking	7	5	2	0.798	6	3	3	0.403
Smoking status, ever	37	19	18	**0.035**	32	12	20	0.387
Heavy smoking	18	6	12	**0.002**	15	7	8	0.255
First degree FHC	25	18	7	0.451	21	11	10	0.138
Diabetes mellitus	24*	15	9	0.548	19	6	13	0.829
**F-PNET**	**n**	**I** + **II (n** = **230)**	**III** + **IV (n** = **13)**	**P**#	n**	**G2** + **G3 (n** = **66)**	**G1 (n** = **153)**	**P**#
Urban area, yes	199	189	10	0.998	188	57	131	0.885
Middle school or higher, yes	224	211	13	0.607	209	62	147	0.754
Alcohol drinking, ever	36	33	3	0.417	32	8	24	0.493
Heavy drinking	27	25	2	0.643	23	9	13	0.246
Smoking status, ever	53	49	4	0.488	50	17	33	0.498
Heavy smoking	26	24	2	0.636	26	10	16	0.324
First degree FHC	29	26	3	0.192	25	6	19	0.477

^*^This was based on only non-functional PNET patients, which included ENETS I + II (n = 93) and ENETS III + IV (n = 49) and included G1 (n = 86), G2 (n = 39) and G3 (n = 17).

^**^There were 32 patients with no information on WHO classification.

^#^Pearson’s *χ*2 test.
