# Supplementary material for: Risk Factors for Sporadic Pancreatic Neuroendocrine Tumors: A Case-Control Study
Source: Sci Rep. 2016 Oct 26;6:36073. doi: 10.1038/srep36073 (PMC5080551; doi:10.1038/srep36073)
Supplement: Supplementary Information [file srep36073-s1.pdf]

Risk Factors for Sporadic Pancreatic Neuroendocrine Tumors: A Case-Control Study

Qiwen Ben[1*], Jie Zhong[1*], Jian Fei[2], Haitao Chen[3], Lifen Yv[1], Jihong Tan[1], Yaozong Yuan[1]

[1] Department of Gastroenterology, Ruijin Hospital, Shanghai Jiaotong University, Shanghai, 200025, China

[2] Department of General Surgery, Ruijin Hospital, Shanghai Jiaotong University, Shanghai, 200025, China

[3]Department of Geriatrics, Changhai Hospital of Second Military Medical University, Shanghai, China

Supplementary Table: Effects of regions and educational levels on the ENETS staging of sporadic pancreatic endocrine tumors according to smoking status

	**Ever Smoking**				**Heavy smoking**		
**PNET**	**I + II (n=68)**	**III + IV (n=22)**	**P#**		**I + II (n=30)**	**III + IV (n=14)**	**P#**
**Region**			**0.530**				**0.256**
Urban area	51	15			16	10	
Rural area	17	7			14	4	
**Education levels**			**0.932**				**0.292**
Elementary school or less	6	2			6	1	
Middle or high school	41	14			18	9	
College or higher level of education	21	6			6	4	
**NF-PNET**	**I + II (n=19)**	**III + IV (n=18)**	**P#**		**I + II (n=6)**	**III + IV (n=12)**	**P#**
**Region**			**0.305**				**0.515**
Urban area	11	14			6	9	
Rural area	8	5			0	3	
**Education levels**			**0.515**				**0.892**
Elementary school or less	2	2			0	1	
Middle or high school	11	9			4	6	
College or higher level of education	6	7			2	5	

#, Pearson's $\chi 2$ test.
